# Fibrocytes in health and disease

**DOI:** 10.1186/1755-1536-5-S1-S6

**Published:** 2012-06-06

**Authors:** Adriana Blakaj, Richard Bucala

**Affiliations:** 1 Department of Medicine, Pathology, Epidemiology and Public Health, Yale University School of Medicine, The Anlyan Center, S525, P.O. Box 208031, 300 Cedar Street, New Haven, CT 06520-8031, USA

## Abstract

Fibrocytes, a group of bone marrow-derived mesenchymal progenitor cells, were first described in 1994 as fibroblast-like, peripheral blood cells that migrate to regions of tissue injury. These cells are unique in their expression of extracellular matrix proteins concomitantly with markers of hematopoietic and monocyte lineage. The involvement of fibrocytes and the specific role they play in the process of wound repair has been a focus of study since their initial description. Fibrocytes contribute to the healing repertoire via several mechanisms; they produce a combination of cytokines, chemokines, and growth factors to create a milieu favorable for repair to occur; they serve as antigen presenting cells (APCs); they contribute to wound closure; and, they promote angiogenesis. Furthermore, regulatory pathways involving serum amyloid P, leukocyte-specific protein 1, and adenosine A_2A _receptors have emphasized the significant role that fibrocytes have in wound healing and fibrosis. The therapeutic targeting of fibrocytes holds promise for the augmentation of wound repair and the treatment of different fibrosing disorders.

## 

The first study of fibrocytes was performed in a murine model of wound repair, which relied on surgical implantation of wound chambers in subcutaneous tissues. Subsequent examination of peripheral blood cells within two days of wound chamber implantation revealed a fibroblast-like circulating CD34^+ ^and Col I^+ ^cell that was in the exudate fluid--adherent and spindle-shaped--and thus termed a fibrocyte [1].

Since their original description, the wealth of information regarding fibrocyte biology has grown steadily and implicated this cell in wound healing and in many disease states that can be regarded as aberrant wound healing, including hypertrophic scars and keloids, airway remodeling in asthma, interstitial pulmonary fibroses, systemic fibroses, atherosclerosis, and the stromal response to tumor invasion [2,3].

Phenotypic examination of these cells reveals a unique cytokine and chemokine profile distinct from monocytes, dendritic cells, Langerhans cells, T lymphocytes, fibroblasts, endothelial cells, and epithelial cells. Fibrocytes express fibroblast components such as vimentin, collagen I, collagen III, and fibronectin, and they express the hematopoietic stem cell marker (CD34) and the leukocyte common antigen (CD45) [1]. Moreover, fibrocytes exhibit unique cytoplasmic extensions that are intermediate in size between microvilli and pseudopodia [1]. Fibrocytes comprise approximately 0.5% of non-erythrocytic cells in the peripheral blood, and they differentiate from CD14+ cells in culture into a phenotype with wound healing potential.

Initial observations of their recruitment to wound sites were found to be due to specific chemokine-chemokine receptor interactions. Namely, it was shown that fibrocytes migrate to wounds in response to secondary lymphoid chemokine (SLC), which is the ligand for CCR7 [4]. The fibrocyte chemokine expression profile also has revealed other chemokine receptors on the surface of fibrocytes, such as CCR3, CCR5, CCR7, and CXCR4 [4], which also likely function in fibrocyte signaling and trafficking pathways.

Fibrocytes produce a combination of cytokines, chemokines, and growth factors that create a milieu favorable for repair. In cutaneous wounds, the CD34 hematopoietic marker initially expressed by fibrocytes decreases over time. This decrease in CD34 occurs in concert with the increased expression of prolyl-4-hydroxylase, an enzyme that is necessary for the stabilization of the collagen triple helix [5]. It is hypothesized that CD34 expression reflects the inflammatory state of the wound, which is down regulated as fibrocytes differentiate into a more mature connective tissue cell [5]. The leukocyte common antigen (CD45) displays a similar decrease in expression as fibrocytes differentiate [6]. This phenotypic transition of fibrocytes is also evident in the context of an increase in expression of TGF-β1 in the wound [5]. TGF-β1, a cytokine that plays an important role in tissue repair and fibrosis, was found to accelerate fibrocyte differentiation to cells that are phentoypically similar to mature fibroblasts and myofibroblasts, *in vitro *[4]. Furthermore, fibrocytes were found to be an important source of cytokines and type I collagen during the inflammatory and repair phase of wound healing. High levels of IL-1β were observed to induce the fibrocyte secretion of chemokines (MIP-1α, MIP-1β), fibrogenic cytokines including TNFα, and hematopoietic growth factors (M-CSF, IL-6, IL-10) [7].

Fibrocytes are potent antigen presenting cells that are capable of initiating and promoting T cell immunity. They constitutively express surface proteins required for antigen presentation including class II major histocompatability complex molecules (HLA-DP, HLA-DQ, and HLA-DR) and the costimulatory molecules CD80 and CD86 [8]. In addition, fibrocytes express the adhesion molecules CD11a, CD54 and CD58 [8]. Furthermore, human fibrocytes were found to induce a tetanus-toxoid specific T cell response that was greater than that induced by peripheral blood monocytes and equivalent to that produced by dendritic cells [8]. In confirmation of in vitro studies, in vivo studies showed that mouse fibrocytes pulsed with foreign antigen and injected in skin migrate to regional lymph nodes and prime naïve T cells [8]. Fibrocytes, therefore, exhibit a potent antigen presenting capability, indicating their likely role in the initiation of immunity during injury, wound repair, and in fibrotic responses associated with inflammation, such as granulomas and scleroderma.

Wound closure is an essential part of wound healing, in which myofibroblasts play an active role. Fibrocytes have been shown to express alpha smooth muscle actin (αSMA) and contract collagen gels *in vitro*, revealing their potential to differentiate into myofibroblasts and contribute to wound contraction [4]. Indeed, fibrocytes also were found to differentiate to a myofibroblast phenotype *in vivo *and express αSMA, thereby confirming that these circulating cells contribute to a subset of myofibroblasts in wounds and are integral mediators of wound healing [6].

Fibrocytes have been shown to promote angiogenesis, which is a requisite for the development and maintenance of new granulation tissue, allowing wound closure and restoration of tissue integrity. Cultured fibrocytes participate in all aspects of neovascularization. They are involved in the proteolysis of the basement membrane by constitutively secreting extracellular matrix degrading enzymes, namely matrix metalloproteinase 9 (MMP-9), thus allowing for endothelial cell invasion. Fibrocytes are involved in the proliferation, migration and tube formation of endothelial cells *in vitro *by their secretion of vascular endothelial growth factor (VEGF), basic fibroblast growth factor (bFGF), IL-8, platelet derived growth factor (PDGF), and hematopoietic growth factors. Moreover, these results were supported by *in vivo *studies using the Matrigel implant model, where the introduction of fibrocytes promoted the formation of new blood vessels [9]. The pro-angiogenic properties of fibrocytes are clearly vital for wound repair, and these findings may provide therapeutic opportunities in the promotion of wound repair.

In order to determine whether these unique cells, indeed, had their origins in the bone marrow, experimental murine models of sex-mismatched bone marrow chimera mice were utilized. In this model, female mice, after total body irradiation, received a male whole bone marrow transplant. Further analysis by in situ hybridization of the wounded tissue in these mice, revealed that circulating fibrocytes and fibrocytes isolated from the wounded tissue were male cells from the transplanted donor bone marrow, thus solidifying their hematopoietic origin [6]. In another model, bone marrow from enhanced green fluorescent protein (EGFP) transgenic mice was transplanted into normal mice. The injured skin of this murine model was found to contain numerous EGFP+, bone-marrow-derived cells. Most of these EGFP+ cells expressed CD45 and were phenotypically similar to fibrocytes, further creating a case for the hematopoietic, stem cell origin of fibrocytes [10].

The regulation of fibrocytes is a dynamic process governed by many factors, each harboring their own therapeutic potential. The profibrotic cytokines, IL-4 and IL-13, along with platelet-derived growth factor (PDGF) promote the differentiation of CD14+ precursors to fibrocytes [11]. Furthermore, the pro-inflammatory cytokines IL-12 and IFN-γ, along with serum amyloid P, and aggregated IgG inhibit the maturation of CD14+ precursors into fibrocytes [11-13]. The subsequent differentiation of fibrocytes to mature mesenchymal cells is stimulated with TGF-β1, as stated previously, as well as with endothelin-1 (ET-1), a peptide that plays an important role in vascular homeostatis [14]. Moreover, fibrocytes can diferentiate into adipocytes when cultured in adipogenic conditions [15], indicating the potential of this cell to differentiate into more than one mesenchemyal cell type.

Recent studies implicating leukocyte-specific protein 1 (LSP1), adenosine A_2A _receptors, and serum amyloid P (SAP) in fibrocyte regulation have further elucidated the role that fibrocytes play, specifically, in the process of wound repair, and they also provide promising therapeutic potential.

LSP1, a cytoskeletal protein reported to be important for leukocyte chemotaxis, may also play a regulatory role in wound healing and fibrocyte biology. Both leukocytes and fibrocytes express LSP1, however fibroblasts do not. Moreover, LSP1 expression persists in fibrocytes and is more upregulated in fibrocytes than it is in leukocytes [16]. Interestingly, LSP1^-/- ^null mice show accelerated healing of skin wounds, along with enhanced collagen synthesis, re-epithelialization, and angiogenesis. Furthermore, the number of fibrocytes in these mice is substantially higher than in wild type mice; and thus, the accelerated wound healing observed in LSP1^-/- ^mice could, partly, be associated with elevated fibrocyte levels [17]. In using this same mouse model and injecting bleomcyin to induce a fibrotic response, the numbers of fibrocytes in LSP1 deficient mice were increased in comparison to WT. Fibrocytes therefore, are not only implicated in the increased fibrosis that is noticed with these mice, but they also are likely a result of an increased mononuclear infiltrate [18]. Thus, elevated fibrocyte levels may accelerate wound healing as well as contribute to pathologic fibrosis.

Adenosine has long been associated with a role in the inflammatory response, which is important for wound healing. It has been shown that adenosine A2 receptor agonists play a beneficial role in wound healing in normal and diabetic animals [19]. Recently, it has become evident that adenosine A_2A _receptors may function in the proliferation and differentiation of mouse bone marrow-derived mesenchymal stem cells (BM-MSCs) [20]. Consistent with these results, the deletion or the blockade of adenosine A_2A _receptors results in less fibrocyte accumulation in the dermis of a bleomycin-treated murine model of scleroderma. Fibrocyte accumulation, therefore, is somehow regulated by adenosine A_2A _receptors [21]. Whether fibrocytes are directly recruited by adenosine A_2A _receptors to tissue, or whether the adenosine A_2A _receptors stimulate the differentiation of precursors to fibrocytes, remains to be further studied. However, the implication that adenosine A_2A _receptors may represent a therapeutic target for treating fibrosing diseases is an exciting one.

SAP, which is a member of the pentraxin family of proteins, is a critical factor in plasma that inhibits fibrocyte differentiation from monocytes *ex vivo *[12]. In a recent study, it has been shown that the administration of exogenous SAP, either locally or systemically, compromises dermal wound healing [22]. SAP-treated wounds showed a decreased rate of wound closure and a decreased number of myofibroblasts at 7 days postwounding [22]. These results are most likely a consequence of an inhibition of fibrocyte differentiation into myofibroblasts. SAP, therefore, represents a potential tool to either ameliorate the efficiency of normal wound healing or prevent aberrant wound healing processes through their regulation of fibrocytes.

In general, adult wound healing results in scar formation, typified by a dysregulated reconstitution of the collagen matrix. Hypertrophic scars represent one form of an aberrant and exuberant wound healing process. Fibrocytes were detected in greater numbers in postburn hypertrophic scar tissue as compared to mature scar tissue and normal dermis, therefore fortifying the hypothesis that they are involved in the development and perpetuation of aberrant wound healing [16]. It has been shown that fibrocytes from either burn patients or normal patients have a much lower ability to synthesize collagen in vitro than do dermal fibroblasts. This finding raises the possibility that the primary role of fibrocytes in the healing of burn wounds may not be in deposition of ECM, and may instead be by the regulation of dermal fibroblasts [23]. Dermal fibroblasts, when conditioned with medium from burn patient fibrocytes, possess a heightened ability to proliferate and migrate, to express αSMA, and to contract collagen lattices in vitro [23]. Furthermore, the profibrotic factors, TGF-β1 and CTGF (connective tissue growth factor), are produced in greater quantities by the fibrocytes of burn patients, and these cytokines may be integral regulators of fibroblast activity in response to thermal injury [23]. Thus, as indicated by this example, the regulation of fibrocytes in pathologic contexts, also awaits further refinement.

Since their first description in 1994, the biology of fibrocytes has been expanded considerably. In wound repair, they have been shown to produce a combination of cytokines, chemokines, and growth factors necessary for the healing process. In addition, their role in antigen presentation, wound closure, and angiogenesis has been demarcated. Fibrocyte regulation via LSP1, adenosine A_2A _receptors, and SAP has also been shown and awaits further characterization. In general, a more detailed view of how fibrocytes mobilize and the trafficking signals they require, as well as how they differentiate, must be further elucidated. There is currently no FDA-approved therapy for fibrosing diseases, and so, a better understanding of fibrocytes and their regulation marks these bone marrow-derived progenitors as potential therapeutic modalities to not only augment normal wound healing, but also potentially reverse fibrosis in many disease states.

## Competing interests

Prof. Bucala serves on the Scientific Advisory Board of Promedior, Inc. which is endeavoring to develop anti-fibrosis therapies.
